# Osteoporosis and Fragility Fractures in Type 2 Diabetes

**DOI:** 10.1155/2020/9342696

**Published:** 2020-07-14

**Authors:** Iacopo Chiodini, Antonino Catalano, Luigi Gennari, Agostino Gaudio

**Affiliations:** ^1^Unit for Bone Metabolism Diseases and Diabetes and Lab of Endocrine and Metabolic Research, IRCCS Instituto Auxologico Italiano, Milan, Italy; ^2^Department of Medical Science and Community Health, University of Milan, Milan, Italy; ^3^Department of Clinical and Experimental Medicine, University of Messina, University Hospital “G. Martino”, Messina, Italy; ^4^Department of Medicine, Surgery and Neurosciences, University of Siena, Policlinico Le Scotte, Siena, Italy; ^5^Department of Clinical and Experimental Medicine, University of Catania, University Hospital “G. Rodolico”, Catania, Italy

Type 2 diabetes mellitus (T2DM) and osteoporosis are associated with severe morbidity, increased mortality, and important social costs, mainly due to their chronic consequences [1]. Epidemiological data indicates that T2DM is associated with increased risk of fractures, suggesting that skeletal fragility should be considered among the chronic complications of T2DM [2, 3] and, in turn, T2DM should be considered among the causes of endocrine osteoporosis [4].

A common feature of the endocrine forms of osteoporosis is the reduced role of bone mineral density (BMD) in predicting fragility fractures [4]. In fact, T2DM does not represent an exception, since, even more than in the other endocrine related osteoporosis forms, it is generally characterised by normal or increased BMD [5]. As a consequence, in T2DM, the risk of fracture is largely independent of BMD, and the latter should not be considered a sensitive enough index of bone fragility [6]. Indeed, if in the presence of reduced BMD an increased risk of fracture has to be considered, then in the presence of normal BMD an increased risk of fractures could not be excluded [5]. Therefore, the fracture risk assessment algorithms, which are significantly based on BMD, are not accurate enough for identifying T2DM patients at risk for fractures [7, 8].

The simpler, but likely incomplete, explanation for the lack of association between BMD and fracture risk in T2DM is that in this endocrine form of osteoporosis reduced bone quality rather than bone density is the main cause of reduced bone strength [9]. This impaired bone quality can be attributed to different mechanisms, whose knowledge represents a challenge for researchers since this information could be used to identify possible targets for both predicting fractures and curing T2DM-related osteoporosis [10].

The reduced bone turnover and impairment of osteoblast activity have been advocated among the possible mechanisms underlying the reduction in bone quality in T2DM [11, 12].

For this special issue, we received different scientific contributions spanning from in vitro studies to animal and clinical research articles.

The paper by Zhang et al., entitled “FOXO1 Mediates Advanced Glycation End Products Induced Mouse Osteocyte-Like MLO-Y4 Cell Apoptosis and Dysfunctions,” explored the capacity of advanced glycation end products (AGEs) to induce osteocyte apoptosis, thus impacting bone homeostasis. Using mouse osteocyte-like MLO-Y4 cells, the authors showed that FOXO1 plays a crucial role in AGE-induced osteocyte dysfunction and apoptosis through its regulation of caspase-3, sclerostin, and RANKL.

In the article by Mohsin et al., entitled “Type 2 Diabetes Mellitus Increases the Risk to Hip Fracture in Postmenopausal Osteoporosis by Deteriorating the Trabecular Bone Microarchitecture and Bone Mass,” using a micro-CT, the authors analysed the changes in the trabecular bone microstructure due to T2DM at various time points in ovariectomised and nonovariectomised rats. Their data suggest that T2DM negatively affects the trabecular structure of the femoral heads of rats and that these changes are correlated with the T2DM duration, increasing the risk of hip fractures.

In the paper by Guo et al., entitled “Assessment of Risk Factors for Fractures in Patients with Type 2 Diabetes over 60 Years Old: A Cross-Sectional Study from Northeast China,” the authors investigated the prevalence of bone fractures in elderly Chinese subjects (with and without T2DM) and evaluated the risk factors for fractures. In particular, when measuring the heel BMD and the timed “up and go” (TUG), the authors observed that low BMD and slow TUG times were independent risk factors for fractures in non-T2DM patients, while no associations were found in the T2DM population. Patients with T2DM had a higher risk for fractures, even when they had preserved BMD and a short TUG time. Therefore, the authors concluded that TUG and BMD underestimated the risk of fractures in the T2DM population.

The review by C. Eller-Vainicher et al., entitled “Pathophysiology and Management of Type 2 Diabetes Mellitus Bone Fragility,” summarised the complex pathophysiological mechanisms underlying bone fragility in T2DM patients. In the first part of the review, the authors analysed the correct clinical approach for evaluating bone health in T2DM patients beyond dual X-ray densitometry, with particular attention to other imaging techniques that have been investigated in recent years, such as trabecular bone score, hip structural analysis, quantitative ultrasound, and peripheral quantitative computed tomography. Moreover, the authors examined the role of microindentation and bone turnover markers in the evaluation of bone fragility in T2DM patients. The second part of the review was dedicated to the factors that lead to bone fragility in T2DM, from disease duration, insulin use, glycometabolic control, and complications to the effects of the different antidiabetic drugs on bone and other metabolic aspects, such as obesity and cortisol secretion.

Finally, the article by Zhao et al., entitled “Association between Uric Acid and Bone Mineral Density in Postmenopausal Women with Type 2 Diabetes Mellitus in China-A cross-sectional Inpatients Study,” retrospectively evaluated the association between uric acid levels and BMD in 262 postmenopausal women with T2DM. The authors concluded that uric acid levels were neither a protective factor nor a risk factor for osteoporosis in these subjects.

We think that the articles in this special issue contribute to increasing the knowledge of the pathogenesis of bone fragility in T2DM patients and may be the starting point for future research.
